# Eosinophilic esophagitis and its association with food allergies: A United States national analysis 2016 to 2022

**DOI:** 10.1371/journal.pone.0335078

**Published:** 2025-10-23

**Authors:** Misha Shah, Abhin Sapkota, Ishaan Aravindaksha, Gedion Yilma Amdetsion, Kriti Katwal, Anas Almoghrabi

**Affiliations:** 1 Department of Internal Medicine, John H. Stroger Jr. Hospital of Cook County, Chicago, Illinois, United States of America; 2 Michigan State University, East Lansing, Michigan, United States of America; 3 Lumbini Medical College, Palpa, Nepal; 4 Division of Gastroenterology, John H. Stroger Jr. Hospital of Cook County, Chicago, Illinois, United States of America; Srebrnjak Children's Hospital, CROATIA

## Abstract

**Background:**

Eosinophilic esophagitis (EoE) has known associations with allergic conditions. Previous studies have reported an increased frequency of EoE in patients with IgE-mediated food allergies (FA), however, statistical validation in large inpatient cohorts remains limited.

**Methods and Findings:**

A retrospective analysis was performed using the National Inpatient Sample (NIS) database from 2016 to 2022. International Classification of Diseases, 10th Revision (ICD-10) codes were used to identify patients with a primary or secondary diagnosis of EoE and FA. Multivariable logistic regression was performed to assess the relationship between EoE and FA. Multivariable regression analysis showed a strong association between EoE and milk (aOR: 7.52, p < 0.001), egg (aOR: 4.77, p < 0.001), peanut (aOR: 3.94, p < 0.001), and seafood allergy (aOR: 2.57, p < 0.001). Among patients with a FA studied, Black (aOR: 0.47, p = 0.001) and Hispanic (aOR: 0.45, p = 0.001) patients had lower odds of EoE compared to White patients. Patients aged 45–64 (aOR: 0.18, p = 0.001) and over 65 (aOR: 0.06, p = 0.001) years with FA had decreased odds of EoE compared to patients under 18. Patients in the highest household zip code income quartile (≥ $86,000) had the greatest odds of EoE (aOR: 1.79, p = 0.001) when compared to the lowest quartile.

**Conclusions:**

Our study supports a strong association between EoE and milk, egg, peanut, and seafood allergies, and the findings across demographics show notable disparities.

## Introduction

Eosinophilic esophagitis (EoE) is a chronic, immune-mediated esophageal disorder with shared pathophysiologic mechanisms with IgE-mediated food allergy (FA). FA are immune-driven hypersensitivities, characterized by the onset of symptoms such as hives, inflammation, and anaphylaxis following exposure to a particular allergen [1]. Both conditions involve a type 2 helper cell (Th2) mechanism triggered by select allergens the body may be hypersensitized to. In addition to this, eosinophils, mast cells, basophils, and Th2-driven cytokines such as IL-4, IL-5, and IL-13 also contribute to the pathogenesis of EoE [2]. Triggers of EoE most commonly include FA and environmental triggers, such as aeroallergens [3,4]. Exposure to triggers of EoE can lead to the recruitment and subsequent accumulation of eosinophils in the esophagus, which results in characteristic esophageal changes found in endoscopy of EoE patients [2,5]. These changes include fixed or transient esophageal rings, linear or longitudinal furrows, and pale mucosa [5]. Additionally, EoE is closely associated with several other immunologically similar diseases, such as atopic dermatitis, asthma, and allergic rhinitis [6].

There is a working case in favor of EoE being a form of chronic FA itself [1,7,8]. Studies have proposed EoE as another form of FA based on similarities in its causes of recurrence, common causation by food antigens, the shared Th2 immune pathway, and treatment methods [1,7,8]. Findings highlight that, due to mechanistic similarities, treatments for EoE could be found in those effective for FAs [1]. Dietary modification is the primary non-pharmacologic approach for the management of EoE [9]. Aside from dietary intervention, oral corticosteroids, esophageal dilation, and proton pump inhibitors are common treatments to manage EoE [10,11]. Despite their popularity, oral steroids may not be efficacious over the long term, mainly due to their side effects and longevity of results [12].

Current literature has documented an increased prevalence of EoE in patients with food allergies [13,14]. However, investigation of the relationship between specific food allergens in EoE within a large inpatient cohort remains limited. This study aims to evaluate the association of specific FA (milk, egg, peanut, and seafood allergy) and the likelihood of EoE across various demographics using the National Inpatient Sample (NIS) database.

## Materials and methods

### Data source

A retrospective cohort analysis was conducted using the NIS from 2016 to 2022. The NIS is a cross-sectional database of US hospitalizations from the Healthcare Cost and Utilization Project (HCUP) from the Agency of Healthcare Research and Quality. The NIS is the largest publicly available, all-payer inpatient care database in the United States. The NIS database has de-identified discharge information from January to December for each given year. The NIS employs a stratified sampling design with discharge weights to produce nationally representative estimates of all US hospitalizations. The data was accessed on 02/01/2025 and none of the authors had access to information that could identify individual discharge information. This study was deemed exempt by the Cook County Health Institutional Review Board as the NIS contains de-identified, publicly available data. Informed consent was waived.

### Study variables

Patients with EoE were identified using the International Classification of Diseases, 10th Revision (ICD-10 code) K20.0, as the primary or secondary diagnosis. Among all patients with EoE, ICD codes were used to identify the cohorts with a primary or secondary diagnosis of FA (milk, egg, peanut, and seafood) or diagnosis of anaphylaxis to these FA. To avoid confounding, only patients with a single FA diagnosis (milk, egg, peanut, or seafood) were included. Patients with multiple FA diagnoses were excluded from the analysis. Non-IgE mediated food allergies were non included in this study. The ICD codes include milk allergy (Z91.011, T78.07XA), egg allergy (Z91.012, T78.08XA), peanut allergy (Z91.010, T78.01XA) and seafood allergy (Z91.013, T78.03XA, T78.02XA).

Factors analyzed for EoE patients with either milk, egg, peanut, or seafood allergies include age, age groups (﹤18 years, 18–65 years, and ＞65 years), sex, race (White, Black, Hispanic, and Other), insurance type (Medicare, Medicaid, private insurance, and self-pay), median household income by zip code (Quartile 1 to Quartile 4), hospital region (Northeast, Midwest, South, West), hospital bed size (small: 1–49 beds, medium: 50–99 beds, and large: more than 100 beds), hospital location and teaching status (nonmetropolitan, metropolitan teaching, and metropolitan non-teaching), and the Charlson Comorbidity Index (CCI). CCI is an empirical prediction of 1-year mortality, based on the number and severity of comorbid diseases by using a weighted index [15]. The CCI was categorized into four groups (CCI Category 0, CCI Category 1, CCI Category 2, and CCI Category 3 or more).

### Statistical analysis

Analysis was done using HCUP survey data analysis packages, incorporating NIS-specific variables, including stratum, hospital identifiers, and discharge weights to account for clustering and large survey-weighted data analysis to obtain statistical and variance calculations. These calculations are independent of individual hospital discharge characteristics. Multiple logistic and linear regression analysis was performed among the whole NIS population, with the presence of milk, egg, peanut, and seafood allergies as independent variables and an EoE diagnosis as the dependent variable.

Our analysis used the weighted sample, which includes over 35 million hospitalizations annually. Weighted survey analysis was performed to generate national estimates. Logistic regression was used to assess the adjusted odds of EoE in patients with FA, controlling for demographic and clinical factors.

This analysis was adjusted for age, sex, race, medical comorbidities in the form of CCI, median household income quartile, insurance status, hospital region, hospital bed size, and hospital location/teaching status as independent variables. Adjusted odds ratios were obtained using marginal effects following multiple regression analysis. A p-value of less than 0.05 was considered statistically significant. All analyses were performed using STATA, version 18 (StataCorp, TX).

## Results

### Descriptive analysis

Of the 46,575 patients admitted with EoE, 1,765 (3.79%) had a comorbid milk allergy, 1,535 (3.30%) had a comorbid egg allergy, 1,460 (3.13%) had a comorbid peanut allergy, and 1,185 (2.54%) had a comorbid seafood allergy. The mean age for all EoE patients was 36.40 years [95% confidence interval (CI), 35.60–37.20]. The mean age for EoE patients with milk, egg, peanut, and seafood allergy comorbidity was distinctly lower at 13.71 years [95% confidence interval (CI), 12.13–15.28], 13.16 years (95% confidence interval (CI), 11.79–14.53], 15.02 years [95% confidence interval (CI), 13.39–16.65], and 22.06 years [95% confidence interval (CI) 19.70–24.41], respectively (Table 1). EoE patients with FA comorbidities were disproportionately represented by pediatric males, with most cases occurring under 18 years of age. Compared to the overall EoE cohort (mean age 36 years), patients with milk, egg, and peanut allergies were markedly younger, with mean ages in the early teens. These patients were also more likely to be White, to receive care in non-metropolitan hospitals, and to be covered by private insurance (Table 1).

**Table 1:** Demographics for patients with Eosinophilic esophagitis and food allergies.

Variables	All EOE patients	EOE patients with food allergy			
		Milk Allergy	Egg Allergy	Peanut allergy	Seafood allergy
Total Admissions	46575	1765 (3.79%)	1535 (3.30%)	1460 (3.13%)	1185 (2.54%)
Mean age in years (95% CI)	36.40 (35.60-37.20)	13.71 (12.13–15.28)	13.16 (11.79-14.52)	15.02 (13.39-16.65)	22.06 (19.70-24.41)
**Age**					
﹤18 years	13770	1335 (75.64%)	1210 (78.83%)	1050 (71.92%)	625 (52.79%)
18 to 65 years	25805	390 (22.10%)	310 (20.20%)	380 (26.03%)	515 (43.50%)
＞65 years	7000	40 (2.27%)	15 (0.98%)	30 (2.05%)	44 (3.72%)
Subtotal	46575	1765	1535	1460	1184
**Sex**					
Male	25850	1150 (65.16%)	1040 (67.75%)	965 (66.14%)	730 (61.66%)
Female	20709	615 (34.84%)	495 (32.25%)	494 (33.86%)	454 (38.84%)
Subtotal	46559	1765	1535	1459	1184
**Race**					
White	34255	1090 (66.87%)	895 (62.59%)	845 (62.13%)	665 (60.18%)
Black	4480	265 (16.26%)	220 (15.38%)	235 (17.28%)	280 (25.34%)
Hispanic	3460	155 (9.51%)	175 (12.24%)	140 (10.29%)	90 (8.14%)
Others	2340	120 (7.36%)	140 (9.79%)	140 (10.29%)	70 (6.33%)
Subtotal	44535	1630	1430	1360	1105
**Charlson Comorbidity Index**					
0	20460	890 (50.42%)	640 (41.69%)	580 (39.73%)	395 (33.33%)
1	14585	740 (41.93%)	755 (49.19%)	730 (50.00%)	620 (52.32%)
2	5425	85 (4.82%)	80 (5.21%)	110 (7.53%)	110 (9.28%)
≥ 3	6105	50 (2.83%)	60 (3.91%)	40 (2.74%)	60 (5.06%)
Subtotal	46575	1765	1535	1460	1185
**Hospital region**					
Northeast	8435	400 (22.66%)	324 (21.14%)	320 (21.93%)	240 (20.25%)
Midwest	12985	370 (20.96%)	360 (23.48%)	359 (24.61%)	285 (24.05%)
South	14500	735 (41.64%)	540 (35.23%)	550 (37.70%)	470 (39.66%)
West	10655	260 (14.73%)	309 (20.16%)	230 (15.76%)	190 (16.03%)
Subtotal	46575	1765	1533	1459	1185
**Hospital bed-size**					
Small	8420	270 (15.30%)	175 (11.40%)	205 (14.04%)	145 (12.24%)
Medium	11535	385 (21.81%)	395 (25.73%)	340 (23.29%)	320 (27.00%)
Large	26620	1110 (62.89%)	965 (62.87%)	915 (62.67%)	720 (60.76%)
Subtotal	46765	1765	1535	1460	1185
**Hospital location and teaching status**					
Non-metropolitan	39310	1690 (95.75%)	1445 (94.20%)	1390 (95.21%)	1115 (94.17%)
Metropolitan non-teaching	5635	55 (3.12%)	84 (5.48%)	60 (4.11%)	59 (4.98%)
Metropolitan teaching	1630	20 (1.13%)	5 (0.33%)	10 (0.68%)	10 (0.84%)
Subtotal	46575	1765	1534	1460	1184
**Insurance**					
Medicare	8765	50 (2.99%)	20 (1.37%)	40 (2.84%)	70 (6.09%)
Medicaid	11180	655 (39.22%)	605 (41.30%)	520 (36.91%)	435 (37.83%)
Private insurance	23340	910 (54.49%)	825 (56.31%)	824 (58.48%)	605 (52.61%)
Self-pay	1530	55 (54.49%)	15 (1.02%)	25 (1.77%)	40 (3.48%)
Subtotal	44815	1670	1465	1409	1150
**Median Household Zipcode Income Quartile**					
Quartile 1 ($1-49,999)	9525	345 (10.62%)	280 (9.89%)	274 (9.95%)	260 (11.98%)
Quartile 2 ($50,000-64,999)	10815	325 (10.00%)	340 (12.01%)	335 (12.16%)	260 (11.98%)
Quartile 3 ($65,000-85,999)	24900	990 (30.46%)	860 (30.39%)	660 (23.97%)	570 (26.27%)
Quartile 4 (≥ $86,000)	39300	1590 (48.92%)	1350 (47.70%)	1485 (53.92%)	1080 (49.77%)
Subtotal	84540	3250	2830	2754	2170

### Outcomes

Among patients with a diagnosis of FA studied, female patients had lower odds of EoE compared to males (aOR: 0.52, p < 0.001). Race-based differences were also observed with Black (aOR: 0.47, p = 0.001) and Hispanic (aOR: 0.45, p = 0.001) patients with FAs having lower odds of EoE compared to White patients. Patients aged 45–64 (aOR: 0.18, p = 0.001) and over 65 (aOR: 0.06, p = 0.001) years with food allergies had decreased odds of EoE when compared to patients under 18 (Table 2).

**Table 2:** Results of multivariable analysis representing the association of Eosinophilic esophagitis with demographic factors among those with food allergy.

	Odds Ratio	95% Confidence Interval	p-value
**Age category**			
< 18 years	Referent	﹣	﹣
18–65 years	0.18	0.15-0.23	<0.001
> 65 years	0.06	0.04-0.11	<0.001
**Race**			
White	Referent	﹣	﹣
Black	0.47	0.37-0.59	<0.001
Hispanic	0.45	0.33-0.60	<0.001
Others	0.44	0.32-0.60	<0.001
**Sex**			
Male	Referent	﹣	﹣
Female	0.52	0.43-63	<0.001
**Charlson Comorbidity Index**			
0	Referent	﹣	﹣
1	1.58	1.31-1.90	<0.001
2	1.04	0.75-1.45	0.808
≥ 3	0.40	0.25-0.63	<0.001
**Hospital region**			
Northeast	Referent	﹣	﹣
Midwest	1.71	1.25-2.34	0.001
South	1.33	1.03-1.72	0.028
West	1.13	0.83-1.52	0.436
**Hospital bed size**			
Small	Referent	﹣	﹣
Medium	1.27	0.96-1.69	0.092
Large	1.60	1.23-2.08	<0.001
**Hospital Location and teaching status**			
Non-metropolitan	Referent	﹣	﹣
Metropolitan non-teaching	1.89	0.74-4.81	0.184
Metropolitan teaching	3.59	1.49-8.65	0.004
**Insurance**			
Medicare	Referent	﹣	﹣
Medicaid	2.34	1.41-3.88	0.001
Private insurance	3.10	1.89-5.09	<0.001
Self-pay	1.83	0.92-3.63	0.082
**Median Household Zipcode Income Quartile**			
Quartile 1 ($1-49,999)	Referent	﹣	﹣
Quartile 2 ($50,000-64,999)	1.37	1.04-1.79	0.024
Quartile 3 ($65,000-85,999)	1.67	1.28-2.18	<0.001
Quartile 4 (≥ $86,000)	1.79	1.39-2.31	<0.001

Patients in the highest household zip code income quartile (≥ $86,000) with food allergy comorbidities were also observed to have the greatest odds of EoE (aOR: 1.79, p = 0.001) when compared to the lowest quartile. Additionally, private insurance (aOR: 3.09, p < 0.001) and Medicaid (aOR: 2.34, p < 0.001) patients with food allergy had increased odds of EoE when compared to Medicare patients.

Using multivariable logistic regression, milk, egg, peanut, and seafood allergies were identified as strongly associated with EoE. Milk allergy was associated with the highest adjusted odds of EoE compared to those without milk allergy (aOR: 7.52, p < 0.001). Followed by egg allergy (aOR: 4.77, p < 0.001), peanut allergy (aOR: 3.94, p < 0.001), and seafood allergy (aOR: 2.57, p < 0.001), respectively.

**Fig 1:**
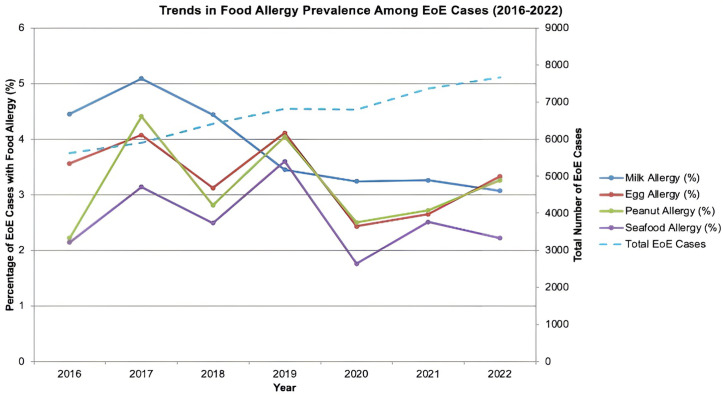
Trends in food allergy prevalence among Eosinophilic esophagitis cases (2016–2022).

From 2016 to 2022, hospital admissions for patients with a primary or secondary diagnosis of EoE increased significantly from 5,620–7,664 (p < 0.001). Among those patients, the prevalence of milk allergy declined from 4.45% in 2016 to 3.07% in 2022, egg allergy from 3.56% to 3.33%, while peanut allergy increased from 2.22% to 3.26%, and seafood allergy from 2.14% to 2.22% (Fig 1 and Table 3). However, these trends were not statistically significant.

**Table 3:** Eosinophilic esophagitis and food allergy trends (2016–2022).

Year	EoE Cases	Milk Allergy	Egg Allergy	Peanut Allergy	Seafood Allergy
2016	5620	250 (4.45%)	200 (3.56%)	125 (2.22%)	120 (2.14%)
2017	5899	300 (5.09%)	240 (4.07%)	260 (4.41%)	185 (3.14%)
2018	6414	285 (4.44%)	200 (3.12%)	180 (2.81%)	160 (2.49%)
2019	6815	235 (3.45%)	280 (4.11%)	275 (4.04%)	245 (3.6%)
2020	6799	220 (3.24%)	165 (2.43%)	170 (2.5%)	120 (1.76%)
2021	7359	240 (3.26%)	195 (2.65%)	200 (2.72%)	185 (2.51%)
2022	7664	235 (3.07%)	255 (3.33%)	250 (3.26%)	170 (2.22%)

## Discussion

Over our study period (from 2016 to 2022), EoE-related hospitalizations increased from 5,620 (95% confidence interval (CI) 5015–6225) in 2016–7,664 (95% confidence interval (CI) 6917–8413) in 2022 (p < 0.001). Prior studies have attributed rising EoE incidence to improved recognition, greater use of esophageal biopsies, and possibly a true increase in prevalence [16,17]. These factors, along with changing environmental exposures, may also explain the increase in EoE-related hospitalizations observed in our analysis. Our findings indicate the rise in EoE cases occurred despite a stable prevalence of milk, egg, peanut, and seafood allergies over the same period (Table 2, Graph 1). Demographic factors also showed a significant role in the likelihood of EoE among patients with FA. Our findings showed that the highest prevalence of patients with EoE and comorbid food allergies was predominantly male. Females with food allergy comorbidities had lower odds of EoE (aOR: 0.52, p < 0.001). This is consistent with previous research where studies have shown EoE is three to four times more common in men than in women [18,19]. Current research also suggests the average age of EoE diagnosis is between 30–50 years [20]. Our data showed the mean ages for EoE patients with milk, egg, peanut, and seafood allergy comorbidity to be significantly younger at 13.71 (95% confidence interval (CI) 12.13–15.28) years, 13.16 (95% confidence interval (CI) 11.79–14.53) years, 15.02 (95% confidence interval (CI) 13.39–16.65) years, and 22.06 (95% confidence interval (CI) 19.70–24.41) years, respectively. Regression analysis demonstrated decreased odds of EoE in patients with comorbid food allergies aged 45–64 years and over 65, compared to patients under 18. These findings underscore the need for earlier screening in high-risk groups, particularly pediatric males with FA. We recommend further investigation into sex and age-related differences in EoE progression, which may help better guide management approach and prevention.

Notably, this study observed the highest prevalence of privately insured patients and those in the highest household income quartile (by zip code) among individuals with food allergies and concurrent EoE. This disparity may reflect barriers to healthcare, differences in healthcare-seeking behavior, or suggest that insurance status influences the likelihood of an EoE diagnosis. This study also observed disparities across race. Our findings showed Black and Hispanic patients with FA comorbidity have significantly lower odds of EoE in comparison to White patients. This pattern may reflect a lower true prevalence of disease or may highlight an underdiagnosis in these populations, as current research suggests food allergy burden disproportionately affects North American racial/ethnic minority populations [21]. Further research is needed to explore the interplay between race, food allergies, and EoE to better improve diagnosis and treatment equity.

Our study provides comprehensive statistical evidence supporting an association between EoE and FAs, particularly milk, egg, peanut, and seafood. Among these, milk allergy had the strongest association with increased odds of EoE. This reinforces the role of cow’s milk as a key trigger in EoE and is consistent with previous research [22,23]. Our findings could support the one-food elimination diet (1FED), which has shown the elimination of cow’s milk to be an effective and acceptable initial dietary therapy for EoE [24,25]. The 1FED has even shown histologic remission in more than 50% of children with EoE with significant improvement in symptoms and endoscopic abnormalities [25,26].

In addition to milk allergy, our study found a lower but still significant association of egg, peanut, and seafood with increased odds of EoE, respectively. This further highlights the shared Th2-driven immune mechanism between the two diseases and the role of multiple food allergies in the pathophysiology of EoE. The presence of multiple food allergies in patients may require a more tailored approach where “step-up” elimination diets (ED) can be used [27]. Step-up diets gradually escalate EDs with targeted eliminations and can be less restrictive than a “top-down” strategy [27,28]. This can help reduce the number of endoscopies patients undergo and the time spent identifying triggers [27,28]. Oral immunotherapy for FA is a recognized risk factor for the development of EoE [8]. However, because the NIS does not capture outpatient therapeutic interventions, we were unable to account for these factors in our analysis. Ultimately, the most effective treatment and ED for EoE is the one that patients can adhere to long-term. We urge future research to focus on optimizing personalized ED approaches and evaluating long-term outcomes using a multidisciplinary approach to enhance overall quality-of-life measures for EoE patients.

A strength of our study is the use of the HCUP NIS as our data source. The NIS is a large, weighted database generalizable to the larger population of food allergy and EoE patients across the United States. A limitation of our study is the potential for inaccuracies in ICD coding at hospitals. This could be due to misclassifications or incomplete documentation of food allergies for patients in the electronic medical record. Additionally, our study centers around patients who have been hospitalized and may not represent patients who are managed with mild to moderate disease in the outpatient setting. Given our limitations, we suggest further randomized controlled trials to contribute to the validity of our results. Future work should also focus on longitudinal cohort studies to clarify causal pathways, further evaluation of EoE risk among patients undergoing oral immunotherapy, and mechanistic investigations into epithelial barrier dysfunction.

## Conclusion

This study provides strong evidence supporting the association between milk, egg, peanut, and seafood allergies with EoE in a large inpatient cohort. These findings may guide clinical decision-making by identifying high-risk patients and support the development of early screening tools and strategies to aid in timely diagnosis and management of patients with EoE and comorbid food allergies.
